# Evaluation of nasogastric tube fixation methods: adhesion, displacement and skin integrity

**DOI:** 10.1590/1518-8345.7411.4365

**Published:** 2024-09-23

**Authors:** Lucia Ingridy Farias Thorpe, Jabiael Carneiro da Silva, Renato Barros Moraes, Nataly da Silva Gonçalves, Alex do Nascimento Alves, Isabel Cristina Ramos Vieira Santos

**Affiliations:** ^1^ Universidade de Pernambuco, Recife, PE, Brazil.; ^2^ Universidade Católica de Pernambuco, Centro de Ciências da Saúde e da Vida, Recife, PE, Brazil.

**Keywords:** Nasogastric Intubation, External Fixators, Patient Safety, Adverse Events, Wounds and Injuries, Nursing Care

## Abstract

**Objective:**

to evaluate three methods of nasogastric tube fixation in terms of adhesion, displacement and skin integrity.

**Method:**

*ex vivo* study, with a sample of 30 experimental noses (10 for each type of fixation), developed with porcine skin, based on the average measurements of the human nose, in which 14-gauge polyvinyl chloride probes were inserted and 2 methods of fixation with adhesive tape (Fixation A and B) and one with an industrial device (Fixation C) were used. Each group was exposed to traction of 50, 100 and 500g sequentially over 12 and 24 hours, testing: adhesion capacity, probe displacement and skin integrity. The Chi-square test of independence was calculated for nominal variables and Student’s t-tests and analysis of variance (p< 0.05) for rational variables.

**Results:**

fixation B showed lower adhesion capacity (p <0.001) when compared to the other two fixations. A mean displacement of 52.17 mm was observed in the probes fixed by methods A and B and a greater occurrence of lesions associated with fixations A and C (p = 0.001).

**Conclusion:**

the results show complications related to the fixations: lack of adhesion, displacement of the probe and skin lesions, drawing attention to the complexity of the procedure.

## Highlights

**(1)** The *ex vivo* experiment was a good research strategy.

**(2)** The commercial fixator showed greater fixation capacity.

**(3)** Both fixator manufacturing techniques were associated with dislocations.

## Introduction

 The nasogastric tube (NGT) is a device commonly used by patients who are unable to receive food, medication and water orally, recommended by the Brazilian Society of Parenteral and Enteral Nutrition (BRASPEn) for short-term procedures. Nasogastric tube feeding is an old therapy, used both in and out of hospital ^(^ ^1^ ^)^ . 

 Although NGT brings benefits, its application is related to risks and possible adverse events (AEs) ^(^ ^2^ ^)^ . Such events are defined as “an unintentional injury that results in temporary or permanent disability and/or prolonged length of stay or death as a consequence of healthcare provided” ^(^ ^3^ ^)^ . 

 Among the main AEs are: accidental removal of the NGT and skin and mucous membrane injuries ^(^ ^4^ ^-^ ^5^ ^)^ . In fact, the occurrence of pressure injuries associated with the use of NGTs, although underestimated, is due to inadequate fixation of the tube, leading to tissue ischemia and skin ulcers. In addition, patients, especially those with an altered mental state, can repeatedly pull on the tube, leading to the risk of reinsertion or malpositioning ^(^ ^6^ ^)^ . 

 Events such as these cause nutritional and medication interruptions and can prolong the patient’s stay in hospital, causing harm to the patient and the institution. In order to prevent such AEs, nursing care must include special attention to securing the tube ^(^ ^7^ ^)^ . 

 According to Decree 94.406/87, which regulates the law on the practice of nursing, the installation of the NGT is the responsibility of the nurse. In addition, nurses are responsible for choosing the method of attachment, monitoring, maintenance and subsequent removal. In this sense, it is important that they develop their practice based on the best evidence, both during insertion and maintenance, and assess the patient’s acceptance of the chosen method of fixation ^(^ ^8^ ^)^ , in order to provide greater comfort during use. 

 NGTs are usually fixed to patients’ skin with adhesive tape or industrialized fixation devices. With regard to fixation using common adhesive tape, a Brazilian author ^(^ ^9^ ^)^ proposed two types of NGS fixation. The first consists of fixing the nasogastric tube with a microporous adhesive tape or adhesive plaster, measuring 13 centimetres’ long by one-centimetre-wide, over the upper lip. 

In the second case, for nutritional purposes, after repeating the above procedures, the tube “is curved upwards and fixed with another adhesive tape, initially wrapped around it, to the back of the nose. Finally, with a third adhesive tape, the tube is attached to the forehead”.

 North American authors ^(^ ^10^ ^)^ , describe a method in which 5 cm of one end of the adhesive tape is split lengthways, the intact end of the tape is placed over the tip of the patient’s nose and each 5cm strip is wrapped around the probe ^(^ ^11^ ^)^ . 

Commercial fastening options, on the other hand, vary in terms of product design, shape, type of adhesive and association with a clamp or lock, although similar characteristics are noticeable.

 In addition to these, other fixing methods are adopted according to each institution. Despite the variety of methods and their frequent use by nurses, the safety of these types of fixation has not been scientifically evaluated ^(^ ^1^ ^)^ and several nursing guidelines related to NGT fixation are not based on evidence, but on rituals and opinions ^(^ ^12^ ^)^ . 

Considering the scarcity of literature on nasogastric tube fixation, this study aimed to evaluate fixation methods in terms of adhesion, displacement and skin integrity.

## Method

### Study design

 This is an observational and comparative study ^(^ ^13^ ^)^ , *ex vivo* , an experiment in a controlled environment, outside a living organism, involving isolated tissues ^(^ ^14^ ^)^ . This type of study, normally applied to chemistry and pharmacology, was indicated in this research as it ensures greater control over possible confounding variables. 

 The advantages of the *ex vivo* study correspond to the 3Rs rule, which are replacement, since it replaces the use of animals or human volunteers in experimentation; reduction, related to the smaller sample size; and refinement, due to the processes used to get closer to reality ^(^ ^15^ ^)^ . 

### Study site

The study was carried out at the Biophysics laboratory of the Catholic University of Pernambuco, from March to July 2022.

### Material for the experiment

 The material for the experiment consisted of: 06 sheets of plywood with the following dimensions: 30x40 cm and 10 mm thick; 06 cuts of pig skin ( *Sus scrofa domesticus* ) measuring 150x130 mm; 1 kg of nails with a 0.9x9 mm gauge head; 1 roll of 100% cotton thread; 30 units of 14-gauge nasogastric tube (Levine tube); 1 unit of 10 cm x 4.5 m waterproof adhesive plaster made up of: cotton with waterproofing acrylic resin and adhesive paste based on natural rubber, zinc oxide and resin for fixing the two techniques (A and B); 10 units of 3M nasal tube and probe fixative, composed of: polyurethane laminated on polyester non-woven, hypoallergenic acrylic adhesive with siliconized paper liner (Fixative C); 1 roll of nylon monofilament 0.30 mm x 100 m, 3 calibration weights for Mettler-Toledo stainless steel digital scales, 50, 100 and 500 g respectively; digital pachymeter with measuring range: 150 mm /0-6” inches and resolution of: 0. 01 mm /0.0005 inch. 

 Two plywood display boards were placed on each bench measuring 170 cm x 80 cm x 50 cm, aligned next to each other. Each experiment group corresponded to two display boards as follows: Group A: fixative A ^(^ ^10^ ^)^ , Group B: fixer B ^(^ ^9^ ^)^ and Group C: Fixative C (commercial fixative for nasal tubes and probes). 

**Figure 1:**
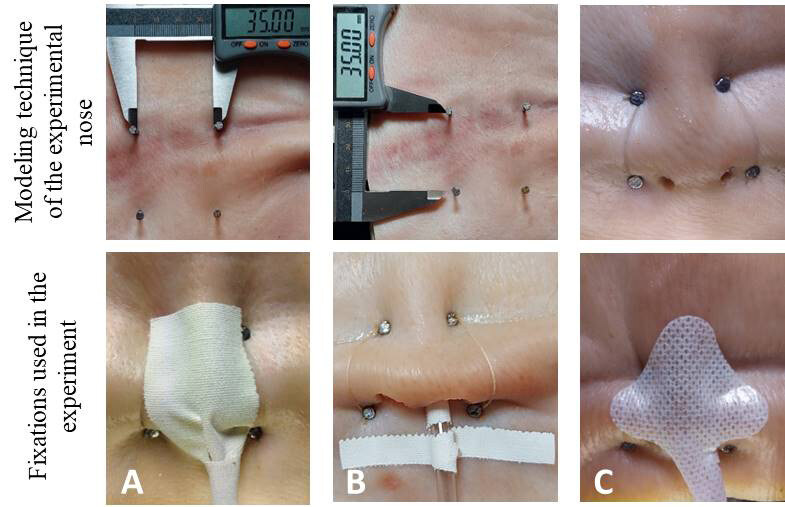
- Modeling technique of the experimental nose and NGT fixations used in the experiment. Recife, PE, Brazil, 2022

 A cut of pork skin was placed on each plate, with the posterior part (fat) facing downwards. Five noses were then constructed per cut (n=30), according to the average nasal measurements for ages 31-40: nasal length (n-prn) of 4.82 cm and 4.58 cm, nasal height (n-sn): 5.22 cm and 4.97 cm and nasal width (al-al): 3.51 cm and 3.10 cm, respectively, for males and females ^(^ ^16^ ^)^ ( Figure 1 ). The experimental nose was then modelled and fixed using nails and sewing thread to the respective display boards. 

 For each experimental nose, two holes were drilled, observing the diameter of the human nostril of 10 and 12 mm ^(^ ^17^ ^)^ , each experimental nose was separated from the other by a distance of 3.5 mm. 

 A nasogastric tube was inserted into each experimental nose, observing a length of 50.7 cm, the average measure of NGS insertion reported in the literature for the method of insertion from the tip of the nose to the earlobe and from there to the xiphoid appendix of an adult human being ^(^ ^18^ ^)^ . This length was marked with a 1x2 cm strip of waterproof adhesive tape around the probe, aligned with the nostril exit, to serve as a reference (reference standard) to check for displacement. 

 The fixation were applied according to the respective procedures recommended in the literature for fixation A ^(^ ^10^ ^)^ , B ^(^ ^9^ ^)^ and fixative C (commercial). Figure 1 below shows the stages for constructing the experimental nose and the NGT fixation methods used in the experiment: 

Each display plate was covered with a polypropylene cloth of the same size, creating a barrier to prevent the pigskins from being exposed to unwanted insects.

The outer end of each probe was connected by a 5 cm nylon thread to stainless steel weights of 50, 100 and 500 g (Mettler-Toledo), sequentially every 24 hours, in order to simulate the various situations of NGT traction.

### Study variables

 The variables considered in this study were: 1) Probe fixation method, categorized as: fixation A ^(^ ^10^ ^)^ , fixative B ^(^ ^9^ ^)^ and fixation C; 2) Adhesion, defined as the ability of the probe to be fixed in the nose as it was inserted, a dichotomous variable (fixation adhered or not adhered to the skin); 3) Displacement, defined as the difference, in millimetres, between the original measurement of the probe at the reference point and the measurement taken 12 and 24 hours after the probe was inserted. This is a rational variable; 4) Skin integrity, assessed as the presence of a lesion 24 hours after the probe was attached. For this purpose, the NPUAP/EPUAP International Pressure Injury Classification System was used, classifying them according to the level of tissue loss: partial thickness loss of the dermis, presenting as a superficial open ulcer and total thickness loss of the tissue when the subcutaneous fat was visible ^(^ ^19^ ^)^ . This variable was also evaluated by the area of the lesion, measured by the length multiplied by the width and by 3.14 (rational variable) and 5) Time, categorized by the intervals of 12 and 24 hours between the probe being fixed and the occurrence of the events of interest. 

### Data collection

The data collection script was drawn up by the researchers and consisted of the variables described above. Data collection was carried out by one of the researchers.

After 12 and 24 hours of each exposure to the respective weight, the displacement of the NGT in relation to the nostril was checked using a digital calliper, calibrated in millimetres, measuring the distance between the reference mark and the nostril.

At the end of the 24-hour experiment, the fixation was removed by a single researcher using an adhesive remover in the shape of a handkerchief, composed of: Hexamethyldisiloxane, Octamethyltrisiloxane, Cyclopentasiloxane, for atraumatic removal, reducing the force required to remove the adhesive.

 All the lesions were photographed using a DSC-HX300 camera with 50x optical zoom and 20.4 MP (Sony Brazil Ltda.), without the use of a flash, and the wound area was measured according to the protocol systematized in the literature ^(^ ^20^ ^)^ , i.e. the photos were taken by placing a disposable ruler next to the wound in parallel with the healthy skin. To minimize error, the camera lens was oriented parallel to the plane of the wound. A second photograph of all the wounds was then taken to ensure at least one (1) good quality photograph. 

The original photographs were then copied to a new folder, and the file names (JPG format) coded to “A1” to “A10”, and so on, respectively, for each group in the experiment, in order to ensure the blinding of the evaluator, who was a stoma therapist nurse with 40 years’ experience in caring for people with skin lesions, who had no information about the type of fixative used in each group.

The digital photographs were viewed using ImageJ 1.45s software (National Institutes of Health, Rockville, MD).

### Data processing and analysis

The mean and standard deviation (SD) of the displacements and the area of the lesions were used for statistical analysis. The chi-square test of independence was calculated to verify the existence of an association between the types of fixations and adhesion capacity, using the likelihood ratio. The Shapiro-Wilk test (normality: p>0.05) was applied to check the normality of the data distribution. The t-Student test was used to compare the means between the two groups of fixations in terms of NGT displacement, and analysis of variance (ANOVA) was calculated for the three groups of fixations in terms of lesion area. For analysis purposes, a 5% significance level was used.

### Ethical aspects

 Considering the type of study carried out [an *ex vivo* study in which sections of pig skin ( *Sus scrofa domesticus* ) were used outside of a living organism (isolated tissues) to simulate the human nose], an evaluation by an Ethics Committee is not necessary. 

## Results

 In the first 12 hours of using the NGT, with a traction of 50 and 100 g, all the fixations remained adhered, but when subjected to a traction of 500 g there was detachment in 10% of the A fixations and 50% of the B fixations. Similarly, after 24 hours of exposure, the fixations remained adhered with the smaller tractions and, when the NGT was subjected to a traction of 500 g, detachment was observed in 10% of the A fixation and 70% of the B fixation. There was no detachment associated with fixation C when it was exposed to the three weights or at 12 and 24 hours ( Table 1 ). 

There was a higher frequency of detachment when using fixative B compared to the others, increasing the difference even with the longer exposure time. The Chi-square test of independence showed an association between detachment of the fixations and traction applied for 12 [χ2(2) = 9.660; p=0.008] and 24 hours [χ2(2) = 16.076; p<0.001].

**Table 1:** - Association of fixation adhesion when subjected to 500 g of traction for 12 and 24 hours. Recife, PE, Brazil, 2022

**Variable**	**Adhesion 12 hours**		**p-value**	**Adhesion 12 hours**		**p-value** ^*^
	**Yes** (%)	**No** (%)		**Yes** (%)	**No** (%)	
Fixation A	9 (90.0)	1(10.0)		9 (90.0)	1 (10.0)	
Fixation B	5 (50.0)	5 (50.0)	0.008	3 (30.0)	7 (70.0)	<0.001
Fixation C	10 (100.0)	0 (0.0)		10 (100.0)	0 (0.0)	

^*^ p-value = Significance level

With regard to the displacement of the NGT when subjected to traction, there was a similar event to that which occurred with regard to adhesion, i.e. tractions of 50 and 100 g were not able to cause displacement of the NGT in the 12 or 24 hours of observation, only traction of 500 g caused displacement in the 12 hours in 40% of fixations A and B (X: 34.04 mm; SD: 4.8) and displacement in 56.7% of the same devices in the 24 hours of observation (X: 34.78 mm; SD: 4.9).

 Of the three fixations analysed, fixation C showed no displacement even when subjected to 500 g traction. The student’s t-test used to compare the mean displacement of fixtures A and B showed that the variances were equal, as can be seen from the p-values >0.05 for the two exposure periods ( Table 2 ). 

**Figure 2:**
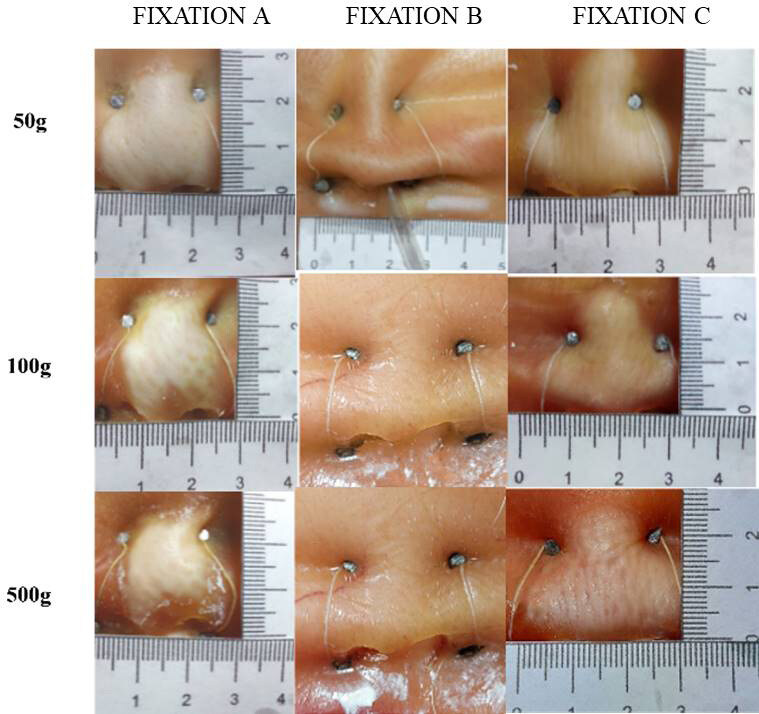
- Lesions according to NGT fixation group. Recife, PE, Brazil, 2022

 As for skin integrity ( Figure 2 ), at the end of the experiment there were lesions on all the noses exposed to the fixation, 90% of which were characterized by total loss of tissue thickness and 10% by partial loss. The highest frequencies of lesions occurred in groups A and C, with a statistically significant difference (p= 0.036). 

**Table 2:** - Comparison of mean displacement between fixations A and B. Recife, PE, Brazil, 2022

**Displacement**	**Fixation**	**n** ^*^	**X mm** ^†^	**SD** ^‡^	**t** ^§^	**p-value** ^||^
12 hours	A	10	72.003	56.3959	1.957	0.066
	B	10	30.120	37.4081		
24 hours	A	10	64.056	46.0694	1.356	0.192
	B	10	40.295	30.7552		

^*^ n = Sample;

^†^ X mm = Mean displacement in millimeters;

^‡^ DP = Standard deviation;

^§^ t = t-Student;

^||^ p-value = Significance level

 The 1-way ANOVA showed that there was an effect of the type of fixation on the lesion area [F (2.27) = 8.88; p: 0.001]. Bonferroni’s *post-hoc* test showed that, on average, the lesion area of the probes fixed with group A differed from those fixed with group B, just as the average lesion area of the probes fixed with group C differed from those fixed with group B ( Table 3 ). 

**Table 3:** - Comparison of mean lesion areas according to nasogastric tube fixation group. Recife, PE, Brazil, 2022

**Groups**	**X** ^*^	**SD** ^†^	**F** ^‡^	**p-value** ^§^
Fixation A	60.43	5.8		
Fixation B	22.90	19.3	8.88	0.001
Fixation C	68.84	40.1		

^*^ X = Average area of lesions;

^†^ SD = Standard deviation;

^‡^ F = Levene’s test;

^§^ p-value = Significance level

## Discussion

Nasogastric probing is an old and commonly performed procedure in people of all ages, whether in home care or in medium or highly complex hospital care, and although it seems like a simple technique, it can be related to serious complications that affect patient safety.

 Adverse events related to the insertion and maintenance stages of the NGS correspond mainly to intrapulmonary placement or migration with the administration of food, medication or fluid; pneumothorax and intra-oesophageal placement or migration, which predisposes to aspiration pneumonia ^(^ ^20^ ^-^ ^21^ ^)^ . 

 Although it is possible to estimate the incidence of adverse events in terms of the number of NGTs, data on the number of tubes used for feeding, hydration, drug administration, gastric lavage and drainage are still unknown in many countries. In the USA, around one million NGS/nasoenteric tubes (NES) are used to treat adults and children every year, and adverse events are estimated to occur in 1-3% of these procedures ^(^ ^21^ ^)^ . The UK has an estimated incidence of 1 adverse event in 10,000 insertions or 0.01% ^(^ ^20^ ^)^ . 

In addition to these, the techniques and/or devices used to fix the nasogastric tube, depending on the length of time it is left in place, the action of gravity and the traction caused both by the connections for washing or draining procedures, and by mechanical action carried out by the patient and/or caregiver themselves, in order to avoid the discomfort caused and/or for cultural reasons, can, in theory, cause it to dislocate, increasing the risk of the events mentioned above.

 This controlled study sought to compare the resistance of three types of fixations, taking as a premise that, despite the existence of industrialized fixations, there is no scientific evidence approving their use and the cost of acquisition is often far from the resources of public health systems. In addition, over time, nurses and other health professionals have developed various fixation techniques, which also lack evidence to support their use ^(^ ^22^ ^)^ . 

 In general, the three types of fixations showed good adhesion capacity. Only fixative B ^(^ ^9^ ^)^ showed inferior performance when exposed to 500 g traction compared to the other two fixation. In this technique, an adhesive tape, measuring 13 centimetres long by one-centimetre-wide, completely wraps around the NGT and is then fixed over the upper lip. The smaller area of the lip and its greater mobility when compared to the nose, at first, seem plausible justifications for this result. 

Fixation C (commercial) showed greater adhesion capacity, preventing displacement of the NGT when compared to the manufactured fixations (fixations A and B), for which no difference was found in terms of average displacement of the NGTs.

 On average, a displacement of 52.17 mm was observed in fixations A ^(^ ^10^ ^)^ and B ^(^ ^9^ ^)^ . The safe length of NGS insertion depends on the measurement technique used and body height, with an average estimate for adults of 55-65 cm, when using the conventional technique, from the nose to the earlobe and from there to the xiphoid appendix ^(^ ^23^ ^)^ . Therefore, the displacement found in our experiment suggests a risk of adverse effects, with possible migration of the probe into the esophagus, which could lead to reflux and bronchoaspiration. 

 The risk of aspiration of gastric contents from the esophagus into the lungs, as well as the association between aspiration pneumonia and esophageal displacement are not known, however, this complication is potentially fatal and should also be the subject of concern regarding the safety of the nasogastric tube procedure ^(^ ^20^ ^)^ . 

 With regard to skin integrity, our study showed that all the fixations tested caused injury with total loss of skin thickness, with higher frequencies for fixation A ^(^ ^10^ ^)^ and fixation C. In fact, both are adhered to the nasal septum, over a considerably larger area than that used in the technique for fixation B. 

 Despite the limited information on injuries related to medical devices, studies have shown a prevalence of 8 to 9.4% of pressure injuries associated with nasogastric tubes ^(^ ^20^ ^,^ ^24^ ^-^ ^25^ ^)^ . The composition of fixation adhesives when exposed to the weather, skin conditions and traction may in theory suggest an explanation for these injuries. 

This study contributes to the advancement of scientific knowledge in the nursing field both because of the method used, which proved useful for testing variables such as adhesion, displacement and the occurrence of skin lesions associated with nasogastric tube fixation, as well as because of the unprecedented results, which allowed for greater reflection on the choice of fixation, the development of protocols and strategies to prevent adverse effects such as skin lesions. However, the limitation is that other variables may influence the interface between the type of fixation and adhesion, displacement and injury, and it is recommended that further in vivo studies be carried out to better elucidate the phenomena described here.

## Conclusion

The results found here are unprecedented and add to knowledge on issues related to nasogastric tube fixations.

Fixation B, developed for greater patient comfort in view of the small fixation area, was associated with adhesion failures, while the commercial fixation (C) showed greater capacity for this.

 Both manufactured fixations were associated with considerable displacement of the NGS, which can expose the patient to a greater risk of adverse events such as gastroesophageal reflux and bronchoaspiration. However, with regard to skin integrity, the results showed that both fixation C and fixation A ^(^ ^10^ ^)^ were associated with total loss of skin thickness. 

The results presented attest to complications related to nasogastric tube fixations and draw attention to the complexity of the procedure. In view of this, nurses’ knowledge and skill when inserting and carrying out subsequent care are important to ensure patient safety.
