# Supplementary material for: Evaluation of nasogastric tube fixation methods: adhesion, displacement and skin integrity
Source: Rev Lat Am Enfermagem. 2024 Sep 23;32:e4365. doi: 10.1590/1518-8345.7411.4365 (PMC11421525; doi:10.1590/1518-8345.7411.4365)
Supplement: Translation

# Translation: Evaluación de los métodos de fijación de sonda nasogástrica: adherencia, desplazamiento e integridad de la piel

**DOI:** 10.1590/1518-8345.7411.4364

**Authors:** Lucia Ingridy Farias Thorpe, Jabiael Carneiro da Silva, Renato Barros Moraes, Nataly da Silva Gonçalves, Alex do Nascimento Alves, Isabel Cristina Ramos Vieira Santos

**Affiliations:** ^1^ Universidade de Pernambuco, Recife, PE, Brasil.; ^2^ Universidade Católica de Pernambuco, Centro de Ciências da Saúde e da Vida, Recife, PE, Brasil.

**Keywords:** Intubación Gastrointestinal, Fijadores Externos, Seguridad del Paciente, Eventos Adversos, Heridas y Lesiones, Atención de Enfermería

## Abstract

**Objetivo:**

evaluar tres métodos de fijación de sonda nasogástrica en cuanto a adherencia, desplazamiento e integridad de la piel.

**Método:**

estudio *ex vivo* , con una muestra de 30 narices experimentales (10 para cada tipo de fijación), desarrolladas con piel porcina, a partir de las medidas medias de la nariz humana, en las cuales se introdujeron sondas de poli cloruro de vinilo calibre 14 y se utilizaron 2 métodos de fijación con esparadrapo (Fijador A y B), y uno con dispositivo industrial (Fijador C). Cada grupo fue expuesto a tracción de 50, 100 y 500g secuencialmente en el período de 12 y 24 horas, testeándose: capacidad de adherencia, desplazamiento de la sonda e integridad de la piel. Se calculó el test Chi-cuadrado de independencia para variables nominales y pruebas t-Student y Análisis de varianza (p< 0,05) para las racionales.

**Resultados:**

el fijador B presentó capacidad inferior de adherencia (p <0,001) cuando comparado a los otros dos fijadores. Se observó un desplazamiento medio de 52,17 mm en las sondas fijadas por los métodos A y B y una mayor ocurrencia de lesiones asociadas a los fijadores A y C (p = 0,001).

**Conclusión:**

los resultados atestiguan complicaciones relacionadas a los fijadores: falta de adherencia, desplazamiento de la sonda y lesiones de piel, llamando atención para la complejidad del procedimiento.

## Destacados

**(1)** El experimento *ex vivo* se constituyó en una buena estrategia de investigación.

**(2)** El fijador comercial mostró una mayor capacidad de fijación.

**(3)** Las dos técnicas de manufactura de fijadores estuvieron asociadas a desplazamientos.

## Introducción

 La sonda nasogástrica (SNG) es un dispositivo comúnmente utilizado por pacientes que están imposibilitados de recibir alimento, medicación y agua por vía oral, recomendado por la *Sociedade Brasileira de Nutrição Parenteral e Enteral* (BRASPEn) para procedimientos de corto plazo. El sondaje nasogástrica es una terapéutica antigua, aplicada tanto en ambiente hospitalario como fuera de él ^(^ ^1^ ^)^ . 

 A pesar de que la SNG trae beneficios, su aplicación está relacionada con riesgos y posibles eventos adversos (EAs) ^(^ ^2^ ^)^ . Tales eventos se definen, “como una lesión no intencional que resulta en incapacidad temporal o permanente y/o prolongación del tiempo de estancia o muerte como consecuencia de un cuidado de salud proporcionado” ^(^ ^3^ ^)^ . 

 Entre los principales EAs están: la remoción accidental de la SNG y lesiones de piel y de mucosa ^(^ ^4^ ^-^ ^5^ ^)^ . De hecho, la ocurrencia de lesión por presión asociada al uso de la SNG, aunque subestimada, se debe a la inadecuada fijación de la sonda, llevando a la isquemia del tejido y úlceras en la piel. Además, pacientes, especialmente con estado mental alterado, pueden traccionar repetidamente la SNG, llevando al riesgo de reinserción o mal posicionamiento ^(^ ^6^ ^)^ . 

 Eventos como estos provocan interrupción nutricional y medicamentosa, y pueden llegar a prolongar el tiempo de permanencia del paciente en ambiente hospitalario, culminando en perjuicio para el mismo y para la institución. A fin de prevenir tales EAs, es necesario que, entre los cuidados de enfermería, haya atención especial respecto a la fijación de la sonda ^(^ ^7^ ^)^ . 

 De acuerdo con el Decreto 94.406/87, que regula la ley del ejercicio de la enfermería, la instalación de la SNG es atribución del enfermero. Además, es él el profesional responsable por la elección del método de fijación, monitoreo, mantenimiento y posterior retirada. En este sentido, es importante que él desarrolle su práctica basada en las mejores evidencias, tanto durante la inserción como en el mantenimiento y evalúe la aceptación del paciente al método de fijación elegido ^(^ ^8^ ^)^ , con el fin de proporcionar mayor confort durante el uso. 

 Comúnmente, las SNGs son fijadas a la piel de los pacientes con cinta adhesiva o dispositivos de fijación industrializados. En cuanto a las fijaciones utilizando cinta adhesiva común, un autor brasileño ^(^ ^9^ ^)^ propuso dos tipos de fijación de SNG. El primero consiste en fijar la sonda nasogástrica con una cinta adhesiva microporosa o de esparadrapo, midiendo 13 centímetros de longitud por un centímetro de ancho, sobre el labio superior. 

Ya en el segundo, con finalidad nutritiva, después de repetidos los procedimientos arriba, la sonda “es curvada hacia arriba y fijada con otra cinta adhesiva, enrollada inicialmente alrededor de ella, al dorso de la nariz. Por último, con una tercera cinta adhesiva, la sonda es presa a la frente”.

 Por su parte, autores norteamericanos ^(^ ^10^ ^)^ , describen un método en el cual se divide 5 cm de una extremidad de la cinta adhesiva en el sentido del largo, colocando la extremidad intacta de la cinta sobre la punta de la nariz del paciente y enrollando cada una de las tiras de 5 cm alrededor de la sonda ^(^ ^11^ ^)^ . 

Ya las opciones de fijación comercial presentan variación en el diseño de los productos en relación a la forma, tipo de adhesivo y asociación con una brazaleta o traba, aunque características similares sean perceptibles.

 Además de estos, otros métodos de fijación son adoptados de acuerdo con cada institución. A pesar de la variedad existente entre los métodos y de la frecuente utilización por los enfermeros, la seguridad de tales tipos de fijación no posee evaluación científica ^(^ ^1^ ^)^ y varias orientaciones de enfermería relacionadas con la fijación de SNG no están basadas en evidencias, sino en rituales y opiniones ^(^ ^12^ ^)^ . 

Considerando la escasez de literatura en cuanto a la fijación de la sonda nasogástrica, este estudio objetivó evaluar los métodos de fijación en cuanto a adherencia, desplazamiento e integridad de la piel.

## Método

### Diseño del estudio

 Se trata de un estudio observacional y comparativo ^(^ ^13^ ^)^ , del tipo *ex vivo* , experimento en ambiente controlado, fuera de un organismo vivo, involucrando tejidos aislados ^(^ ^14^ ^)^ . Este tipo de estudio, normalmente aplicado a la química y farmacología, encontró indicación en esta investigación por asegurar mayor control sobre posibles variables de confusión. 

 Las ventajas del estudio *ex vivo* corresponden a la regla de los 3Rs, que son la sustitución ( *replacement* ), ya que sustituyó el uso de animales o voluntarios humanos en la experimentación; la reducción ( *reduction* ), relacionada con el menor número de la muestra; y el refinamiento ( *refinement* ), debido a los procesos utilizados para mayor aproximación a la realidad ^(^ ^15^ ^)^ . 

### Lugar del estudio

El estudio se realizó en el laboratorio de Biofísica de la Universidad Católica de Pernambuco, en el período de marzo a julio de 2022.

### Material para el experimento

 El material para el experimento consistió en: 06 placas de contrachapado con las siguientes dimensiones: 30x40 cm y 10 mm de espesor; 06 cortes de piel de cerdo ( *Sus scrofa domesticus* ) midiendo 150x130 mm; 1 kg de clavos con cabeza con calibre de 0,9x9 mm; 1 rollo de hilo 100% algodón; 30 unidades de sonda nasogástrica (sonda de Levine) calibre 14; 1 unidad de esparadrapo impermeable de 10 cm x 4,5 m compuesto por: algodón con resina acrílica impermeabilizante y masa adhesiva a base de caucho natural, óxido de zinc y resina para las fijaciones de las dos técnicas (A y B); 10 unidades de 3M fijador de tubos y sondas nasales, compuesto por: poliuretano laminado sobre no tejido de poliéster, adhesivo acrílico hipoalergénico con *liner* de papel siliconado (Fijador C); 1 rollo de monofilamento de nylon 0,30 mm x 100 m, 3 pesos de calibración para balanza digital de acero inoxidable Mettler-Toledo, respectivamente de 50, 100 y 500 g; calibrador digital con rango de medición: 150 mm /0-6” pulgadas y resolución de: 0.01 mm /0.0005 pulgada. 

 En cada banco de 170 cm x 80 cm x 50 cm se colocaron dos placas expositoras de contrachapado, alineadas una al lado de la otra. Cada grupo de experimento correspondió a dos placas expositoras de la siguiente forma: Grupo A: fijador A ^(^ ^10^ ^)^ , Grupo B: fijador B ^(^ ^9^ ^)^ y Grupo C: Fijador C (fijador de tubos y sondas nasales comercial). 

 Sobre cada placa se colocó un corte de piel de cerdo, con la parte posterior (grasa) hacia abajo, construyéndose así cinco narices por corte (n=30), obedeciendo las medidas nasales medias para la edad de 31-40 años: longitud nasal (n-prn) de 4,82 cm y 4,58 cm, altura nasal (n-sn): 5,22 cm y 4,97 cm y anchura nasal (al-al): 3,51 cm y 3,10 cm, respectivamente, para los sexos masculino y femenino ^(^ ^16^ ^)^ ( Figura 1 ). La nariz experimental fue entonces modelada y fijada mediante el uso de clavos e hilo de coser en las respectivas placas expositoras. 

 Para cada nariz experimental se realizaron dos agujeros, observando el diámetro de la narina humana de 10 y 12 mm ^(^ ^17^ ^)^ , utilizando un molde metálico y bisturí con hoja n° 11. Cada nariz experimental fue separada de la otra por una distancia de 3,5 mm. 

 Una sonda nasogástrica fue insertada en cada nariz experimental, observando la longitud de 50,7 cm, medida promedio de inserción de la SNG relatada en la literatura para el método de inserción de la punta de la nariz al lóbulo de la oreja y de este hasta el apéndice xifoides de un adulto humano ^(^ ^18^ ^)^ . Este largo fue marcado con una cinta de 1x2 cm de esparadrapo impermeable alrededor de la sonda, alineada a la salida de la narina, para servir de referencia (marca de referencia) a la verificación de desplazamiento. 

 Los fijadores fueron aplicados de acuerdo con los respectivos procedimientos recomendados por la literatura para los fijadores A ^(^ ^10^ ^)^ , B ^(^ ^9^ ^)^ y del fijador C (comercial). La Figura 1 abajo presenta las etapas para construcción de la nariz experimental y los métodos de fijación de SNG utilizados en el experimento: 

**Figura 1:** - Técnica de modelado de la nariz experimental y fijadores de sonda nasogástrica utilizados en el experimento. Recife, PE, Brasil, 2022

Cada placa expositora fue cubierta por un tejido de polipropileno de la misma dimensión, creando una barrera con el objetivo de evitar la exposición de las pieles de cerdo a insectos indeseables.

La extremidad exterior de cada sonda fue conectada por un hilo de nylon de 5 cm a pesos de acero inoxidable de 50, 100 y 500 g (Mettler-Toledo), secuencialmente, cada 24 horas, de modo a simular las diversas situaciones de tracción de la SNG.

### Variables del estudio

 Las variables consideradas en este estudio fueron: 1) Método de fijación de la sonda, categorizado como: fijador A ^(^ ^10^ ^)^ , fijador B ^(^ ^9^ ^)^ y fijador C; 2) La adherencia, definida como la capacidad de fijación de la sonda en la nariz, en la medida en que fue introducida, variable dicotómica (fijación adherida o no adherida a la piel); 3) El desplazamiento, definido como la diferencia, en milímetros, entre la medida original de la sonda en el punto de referencia y la medida comprobada a las 12 y 24 horas después de la introducción de la sonda. Se trata de una variable racional; 4) La integridad de la piel, evaluada como la presencia de lesión después de 24 horas de la fijación de la sonda. Para ello se utilizó el Sistema Internacional de clasificación de lesiones por presión NPUAP/EPUAP, clasificándolas de acuerdo al nivel de pérdida de tejido en: pérdida parcial de espesor de la dermis, presentándose como úlcera abierta superficial y pérdida total de espesor del tejido cuando la grasa subcutánea era visible ^(^ ^19^ ^)^ . Esta variable también fue evaluada por el área de la lesión, medida por el largo multiplicado por el ancho y por 3,14 (variable racional) y 5) Tiempo, categorizado por los intervalos de 12 y 24 horas entre la fijación de la sonda y la ocurrencia de los eventos de interés. 

### Recolección de datos

El guion de recolección de datos fue elaborado por los investigadores e incluyó las variables anteriormente descritas. La recolección de datos fue realizada por una de las investigadoras.

Después de 12 y 24 horas de cada exposición al respectivo peso, se verificó el desplazamiento de la SNG en relación con la narina, utilizando un calibrador digital, calibrado en milímetros, midiendo la distancia entre la marca de referencia y la narina.

Al final de 24 horas de experimento, se retiró la fijación, por un único investigador, usando un removedor de adhesivo en formato de pañuelo, compuesto por: Hexametildisiloxano, Octametiltrisiloxano, Ciclopentasiloxano, para remoción atraumática, disminuyendo la fuerza necesaria para retirar el adhesivo.

 Todas las lesiones fueron fotografiadas, por una cámara DSC-HX300 con zoom óptico de 50x y 20.4 MP (Sony Brasil Ltda.), sin uso de flash, y la medición del área de la herida fue realizada de acuerdo con el protocolo sistematizado en la literatura ^(^ ^20^ ^)^ , es decir: las fotos fueron tomadas colocando una regla desechable cerca de la herida en paralelo con la piel sana. Para minimizar el error, la lente de la cámara fue orientada paralelamente al plano de la herida. A continuación, se obtuvo una segunda fotografía de todas las heridas para garantizar al menos 1 (una) fotografía de buena calidad. 

Las fotografías originales fueron entonces copiadas a una nueva carpeta, y los nombres de los archivos (formato JPG) codificados para “A1” hasta “A10”, y así respectivamente, para cada grupo del experimento, de modo a garantizar el cegamiento del evaluador, que fue un enfermero estomaterapeuta, con experiencia de 40 años con cuidados de personas con lesión de piel, que no tuvo información acerca del tipo de fijador utilizado en cada grupo.

 Las fotografías digitales fueron visualizadas con el *software* ImageJ 1.45s ( *National Institutes of Health* , Rockville, MD). 

### Tratamiento y análisis de datos

Para el tratamiento estadístico, se utilizaron la media y desviación estándar (DE) de los desplazamientos ocurridos y del área de las lesiones. Se calculó el test Chi-cuadrado de independencia para verificar la existencia de asociación entre los tipos de fijadores y la capacidad de adherencia, mediante la razón de verosimilitud. Para verificar la normalidad de la distribución de los datos, se aplicó el test de Shapiro-Wilk (normalidad: p> 0,05). Para la comparación de promedios entre dos grupos de fijadores en cuanto al desplazamiento de la SNG se utilizó el test t-Student y, el análisis de varianza (ANOVA) se calculó para los tres grupos de fijadores en cuanto al área de la lesión. Para fines de análisis, se observó el nivel de significancia del 5%.

### Aspectos éticos

 Considerando el tipo de estudio realizado [estudio *ex vivo* en el que se utilizaron cortes de piel de cerdo ( *Sus scrofa domesticus)* fuera de un organismo vivo (tejidos aislados) para simular la nariz humana], se dispensa la evaluación por Comité de Ética. 

## Resultados

 En las primeras 12 horas de uso de la SNG, con una tracción de 50 y 100 g, todos los fijadores permanecieron adheridos, pero cuando se sometieron a una tracción de 500 g hubo desprendimiento en el 10% de los fijadores A y en el 50% de los fijadores B. De igual manera, después de 24 horas de exposición, los fijadores se mantuvieron adheridos con las tracciones menores y, cuando la SNG fue sometida a una tracción de 500 g, se observó desprendimiento en el 10% de los fijadores A y en el 70% de los fijadores B. No se observó desprendimiento asociado al fijador C cuando fue expuesto a los tres pesos ni a los tiempos de 12 y 24 horas ( Tabla 1 ). 

Se nota la mayor frecuencia de desprendimiento durante el uso del fijador B en comparación con los otros, aumentando la diferencia, incluso en cuanto al mayor tiempo de exposición. El test Chi-cuadrado de independencia mostró la existencia de asociación entre el desprendimiento de los fijadores en cuanto a la tracción aplicada por 12 [χ2(2)= 9,660; p=0,008] y 24 horas [χ2(2)= 16,076; p<0,001].

**Tabla 1:** - Asociación de la adherencia del fijador en cuanto a la sumisión de 500 g de tracción por 12 y 24 horas. Recife, PE, Brasil, 2022

**Variable**	**Adherencia 12 horas**		**p-valor**	**Adherencia 24 horas**		**p-valor** ^*^
	**Sí** ^(%)^	**No** ^(%)^		**Sim** ^(%)^	**Não** ^(%)^	
Fijador A	9 (90,0)	1(10,0)		9 (90,0)	1 (10,0)	
Fijador B	5 (50,0)	5 (50,0)	0,008	3 (30,0)	7 (70,0)	<0,001
Fijador C	10 (100,0)	0 (0,0)		10 (100,0)	0 (0,0)	

^*^ p-valor = Nivel de significancia

En lo que respecta al desplazamiento de la SNG cuando fue sometida a tracción, se verificó un evento similar al ocurrido en cuanto a la adherencia, es decir, las tracciones de 50 y de 100 g no fueron capaces de causar el desplazamiento de la SNG en las 12 ni en las 24 horas de observación, solo la tracción de 500 g determinó un desplazamiento a las 12 horas en el 40% de los fijadores A y B (X: 34,04 mm; DE: 4,8) y un desplazamiento en el 56,7% de los mismos dispositivos a las 24 horas de observación (X: 34,78 mm; DE: 4,9).

 Entre las tres fijaciones analizadas, la fijación C no presentó desplazamiento incluso cuando fue sometida a una tracción de 500 g. El test t-Student realizado para comparar las medias de desplazamiento de las fijaciones A y B demostró la igualdad de varianzas, conforme se puede verificar por los valores de p >0,05 en los dos períodos de exposición ( Tabla 2 ). 

 En cuanto a la integridad de la piel ( Figura 2 ), al final del experimento se observó la ocurrencia de lesiones en todos las narices expuestas a los fijadores, de los cuales el 90% se caracterizaron por pérdida total de espesor del tejido y el 10% por pérdida parcial. Las mayores frecuencias de lesión ocurrieron para los grupos A y C con diferencia estadística significativa (p= 0,036). 

**Tabla 2:** - Comparación entre los promedios de desplazamiento entre los fijadores A y B. Recife, PE, Brasil, 2022

**Desplazamiento**	**Fijador**	**n** ^*^	**X mm** ^†^	**DE** ^‡^	**t** ^§^	**p-valor** ^||^
Desplazamiento en las 12 horas	Fijador A	10	72,003	56,3959	1,957	0,066
	Fijador B	10	30,120	37,4081		
Desplazamiento en las 24 horas	Fijador A	10	64,056	46,0694	1,356	0,192
	Fijador B	10	40,295	30,7552		

^*^ n = Muestra;

^†^ X mm = Promedio del desplazamiento en milímetros;

^‡^ DE = Desviación-estándar;

^§^ t = t-Student;

^||^ p-valor = Nivel de significancia

**Figura 2:** - Lesiones según el grupo de fijación de la SNG. Recife, PE, Brasil, 2022.

 La ANOVA de una vía mostró que existe un efecto del tipo de fijador sobre el área de la lesión [F (2,27)= 8,88; p: 0,001]. El *post-hoc* de Bonferroni mostró que en promedio el área de la lesión de las sondas fijadas con el grupo A es diferente de aquellas fijadas por el grupo B, así como el promedio del área de la lesión de las sondas fijadas con el grupo C es diferente de las que fueron fijadas por el grupo B ( Tabla 3 ). 

**Tabla 3:** - Comparación de los promedios de las áreas de las lesiones según el grupo de fijación de la sonda nasogástrica. Recife, PE, Brasil, 2022

**Grupos**	**X** ^*^	**DE** ^†^	**F** ^‡^	**p-valor** ^§^
Fijador A	60,43	5,8		
Fijador B	22,90	19,3	8,88	0,001
Fijador C	68,84	40,1		

^*^ X = Promedio de las áreas de las lesiones;

^†^ DE = Desviación-estándar;

^‡^ F = Test de Levene;

^§^ p-valor = Nivel de significancia

## Discusión

El sondaje nasogástrica es un procedimiento antiguo y comúnmente realizado en personas de todas las edades, tanto en la atención domiciliaria como en los cuidados hospitalarios de media o alta complejidad, y aunque parece una técnica simple, puede estar relacionada con complicaciones serias que afectan la seguridad del paciente.

 Los eventos adversos relacionados con la etapa de inserción y mantenimiento de la SNG corresponden principalmente a la colocación o migración intrapulmonar con administración de alimento, medicamento o fluido; neumotórax y colocación o migración intraesofágica, que predisponen a la neumonía por aspiración ^(^ ^20^ ^-^ ^21^ ^)^ . 

 Aunque es posible estimar la incidencia de eventos adversos en términos del número de SNGs, los datos sobre el número de sondas utilizadas para alimentación, hidratación, administración de medicamentos, lavado gástrico y drenaje aún son desconocidos en muchos países. En los EE.UU., cerca de un millón de SNG/Sondas nasoentéricas (SNE) se utilizan en el tratamiento de adultos y niños cada año, y se estima la ocurrencia de eventos adversos en 1 a 3% de esos procedimientos ^(^ ^21^ ^)^ . Inglaterra presenta una incidencia estimada de 1 evento adverso en 10,000 inserciones o 0,01% ^(^ ^20^ ^)^ . 

Además de estos, las técnicas y/o los dispositivos usados para la fijación de la sonda nasogástrica, de acuerdo con el tiempo de permanencia, la acción de la gravedad y la tracción causada tanto por las conexiones para procedimientos de lavado o drenaje, como por acción mecánica, realizada por el propio paciente o cuidador, para evitar el malestar causado y/o por cuestiones culturales, pueden, en teoría, causar su desplazamiento, aumentando el riesgo para los eventos anteriormente citados.

 Sobre todo en cuanto al desplazamiento de la SNG, este estudio controlado buscó verificar, por medio de comparación, la resistencia de tres tipos de fijadores, tomando como premisa que, a pesar de la existencia de fijadores industrializados, no existen evidencias científicas que aprueben su uso y, el costo de adquisición muchas veces está lejos de los recursos de los sistemas públicos de salud. Además, a lo largo del tiempo, enfermeros y otros profesionales de la salud han desarrollado varias técnicas de fijación, que también carecen de evidencias para fundamentar su uso ^(^ ^22^ ^)^ . 

 En general, los tres tipos de fijadores mostraron una buena capacidad de adherencia. Solo el fijador B ^(^ ^9^ ^)^ presentó un rendimiento inferior cuando fue expuesto a una tracción de 500 g, comparado con los otros dos fijadores. En esta técnica, una cinta adhesiva, midiendo 13 centímetros de longitud por un centímetro de ancho, envuelve completamente la SNG y es, a continuación, fijada sobre el labio superior. La menor área del labio y su mayor movilidad en comparación con la nariz, a primera vista, parecen justificaciones plausibles para este resultado. 

El fijador C (comercial) mostró una mayor capacidad de adherencia, evitando el desplazamiento de la SNG en comparación con los fijadores manufacturados (fijador A y B), para los cuales no se encontró diferencia en cuanto a los promedios de desplazamiento de las SNGs.

 En promedio, se observó un desplazamiento de 52,17 mm en las fijaciones A ^(^ ^10^ ^)^ y B ^(^ ^9^ ^)^ . La longitud segura de inserción de la SNG depende de la técnica de medición utilizada y de la altura corporal, estimándose en promedio para adulto 55-65 cm, cuando se utiliza la técnica convencional, de la nariz al lóbulo de la oreja y de esta hasta el apéndice xifoide ^(^ ^23^ ^)^ . Por lo tanto, el desplazamiento encontrado en nuestro experimento sugiere un riesgo para efectos adversos, con posible migración de la sonda al esófago lo que puede ocasionar reflujo y broncoaspiración. 

 El riesgo de aspiración del contenido gástrico del esófago a los pulmones, así como la asociación entre neumonía por aspiración y desplazamiento esofágico no son conocidos, sin embargo, esta complicación es potencialmente fatal y también debe ser motivo de preocupación con la seguridad del procedimiento de sondaje nasogástrico ^(^ ^20^ ^)^ . 

 En cuanto a la integridad de la piel, nuestro estudio demostró que todos los fijadores probados causaron lesión con pérdida total del espesor de la piel, con mayores frecuencias para el fijador A ^(^ ^10^ ^)^ y para el fijador C. De hecho, ambos están adheridos al septo nasal, en un área considerablemente mayor que la utilizada en la técnica por el fijador B. 

 A pesar de la limitada información acerca de lesiones relacionadas con dispositivos médicos, estudios han demostrado una prevalencia de 8 a 9,4% de las lesiones por presión asociadas a sonda nasogástrica ^(^ ^20^ ^,^ ^24^ ^-^ ^25^ ^)^ . La composición de los adhesivos de los fijadores cuando están expuestos al tiempo, las condiciones de la piel y la tracción pueden teóricamente sugerir una explicación para estas lesiones. 

El presente estudio contribuye al avance del conocimiento científico en el área de enfermería tanto por el método utilizado, que se mostró útil para probar variables como adherencia, desplazamiento y ocurrencia de lesión de piel asociadas a la fijación de sonda nasogástrica, así como también por los resultados inéditos que permitieron mayor reflexión para la elección de la fijación, elaboración de protocolos y de estrategias de prevención de efectos adversos como la lesión de piel. Sin embargo, se asume como limitación el hecho de que otras variables pueden influir en la interfaz entre el tipo de fijación y la adherencia, desplazamiento y lesión, recomendándose que nuevos estudios, in vivo, se realicen para mejor elucidar los fenómenos aquí descritos.

## Conclusión

Los resultados aquí encontrados son inéditos y añaden al conocimiento sobre cuestiones relacionadas con los fijadores de sonda nasogástrica.

El fijador B, desarrollado para mayor confort del paciente, teniendo en cuenta la pequeña área de fijación, estuvo asociado a fallos de adherencia, mientras que el fijador comercial (C) mostró una mayor capacidad para esto.

 Los dos fijadores manufacturados estuvieron asociados a desplazamientos considerables de la SNG, lo que puede exponer al paciente a un mayor riesgo de eventos adversos como reflujo gastroesofágico y broncoaspiración. Sin embargo, en lo que respecta a la integridad de la piel, los resultados demostraron que tanto el fijador C como el fijador A ^(^ ^10^ ^)^ estuvieron asociados a lesiones de pérdida total del espesor de la piel. 

Los resultados presentados certifican complicaciones relacionadas con los fijadores de la sonda nasogástrica y llaman la atención sobre la complejidad del procedimiento. Ante esto, el conocimiento y habilidad del enfermero en la inserción y en la realización de los cuidados subsecuentes son importantes para garantizar la seguridad del paciente.

# Translation: Avaliação dos métodos de fixação de sonda nasogástrica: adesão, deslocamento e integridade da pele

**DOI:** 10.1590/1518-8345.7411.4366

**Authors:** Lucia Ingridy Farias Thorpe, Jabiael Carneiro da Silva, Renato Barros Moraes, Nataly da Silva Gonçalves, Alex do Nascimento Alves, Isabel Cristina Ramos Vieira Santos

**Affiliations:** ^1^ Universidade de Pernambuco, Recife, PE, Brasil.; ^2^ Universidade Católica de Pernambuco, Centro de Ciências da Saúde e da Vida, Recife, PE, Brasil.

**Keywords:** Intubação Nasogástrica, Fixadores Externos, Segurança do Paciente, Eventos Adversos, Ferimentos e Lesões, Cuidados de Enfermagem

## Abstract

**Objetivo:**

avaliar três métodos de fixação de sonda nasogástrica quanto à adesão, deslocamento e integridade da pele.

**Método:**

estudo *ex vivo* , com uma amostra de 30 narizes experimentais (10 para cada tipo de fixação), desenvolvidos com pele suína, a partir das medidas médias do nariz humano, nos quais foram introduzidas sondas de poli cloreto de vinila calibre 14 e utilizados dois métodos de fixação com esparadrapo (Fixador A e B), e, um com dispositivo industrial (Fixador C). Cada grupo foi exposto à tração de 50, 100 e 500g sequencialmente no período de 12 e 24 horas, testando-se: capacidade de adesão, deslocamento da sonda e integridade da pele. Calculou-se o teste Qui-quadrado de independência para variáveis nominais e testes t-Student e Análise de variância (p< 0,05) para as racionais.

**Resultados:**

o fixador B apresentou capacidade inferior de adesão (p <0,001) quando comparado aos outros dois fixadores. Observou-se um deslocamento médio de 52,17 mm nas sondas fixadas pelos métodos A e B e uma maior ocorrência de lesões associadas aos fixadores A e C (p = 0,001).

**Conclusão:**

os resultados atestam para complicações relacionadas aos fixadores: falta de adesão, deslocamento da sonda e lesões de pele chamando atenção para complexidade do procedimento.

## Destaques

**(1)** O experimento *ex vivo* se constituiu em boa estratégia de pesquisa.

**(2)** O fixador comercial apresentou maior capacidade de fixação.

**(3)** As duas técnicas de manufatura de fixadores estiveram associadas a deslocamentos.

## Introdução

 A sonda nasogástrica (SNG) é um dispositivo comumente utilizado por pacientes que estão impossibilitados de receber alimento, medicamentos e água por via oral, recomendadas pela Sociedade Brasileira de Nutrição Parenteral e Enteral (BRASPEn) para procedimentos de curto prazo. A sondagem nasogástrica é uma terapêutica antiga, aplicada tanto em ambiente hospitalar quanto fora dele ^(^ ^1^ ^)^ . 

 Apesar da SNG trazer benefícios, sua aplicação está relacionada a riscos e possíveis eventos adversos (EAs) ^(^ ^2^ ^)^ . Tais eventos são definidos, “como uma lesão não intencional que resulte em incapacidade temporária ou permanente e/ou prolongamento do tempo de permanência ou morte como consequência de um cuidado de saúde prestado” ^(^ ^3^ ^)^ . 

 Entre os principais EAs estão: a remoção acidental da SNG e lesões de pele e de mucosa ^(^ ^4^ ^-^ ^5^ ^)^ . De fato, a ocorrência de lesão por pressão associada ao uso de SNG, embora subestimada, deve-se à inadequada fixação da sonda, levando à isquemia do tecido e úlceras na pele. Além disso, pacientes, especialmente com estado mental alterado, podem tracionar repetidamente a SNG, levando ao risco de reinserção ou mau posicionamento ^(^ ^6^ ^)^ . 

 Eventos como esses provocam interrupção nutricional e medicamentosa e podem vir a prolongar o tempo de permanência do paciente em ambiente hospitalar, culminando em prejuízo para o mesmo e para a instituição. A fim de prevenir tais EAs, é preciso que, entre os cuidados de enfermagem, haja atenção especial quanto à fixação da sonda ^(^ ^7^ ^)^ . 

 De acordo com o Decreto 94.406/87, que regula a lei do exercício da enfermagem, a instalação da SNG é atribuição do enfermeiro. Além disso, é ele o profissional responsável pela escolha do método de fixação, monitoramento, manutenção e posterior retirada. Nesse sentido, é importante que ele desenvolva a sua prática baseada nas melhores evidências, tanto durante a inserção quanto para a sua manutenção, avaliando a aceitação do paciente ao método de fixação escolhido ^(^ ^8^ ^)^ , com a finalidade de proporcionar maior conforto durante o uso. 

 Comumente, as SNGs são fixadas à pele dos pacientes com fita adesiva ou dispositivos de fixação industrializados. Quanto às fixações utilizando fita adesiva comum, um autor brasileiro ^(^ ^9^ ^)^ propôs dois tipos de fixação de SNG. O primeiro consiste em fixar a sonda nasogástrica com uma fita adesiva microporosa ou de esparadrapo, medindo 13 centímetros de comprimento por um centímetro de largura, sobre o lábio superior. 

Já no segundo, com finalidade nutritiva, após repetidos os procedimentos acima, a sonda “é curvada para cima e fixada com outra fita adesiva, enrolada inicialmente em torno dela, ao dorso do nariz. Por último, com uma terceira fita adesiva, a sonda é presa à testa”.

 Por sua vez, autores norte-americanos ^(^ ^10^ ^)^ descrevem um método no qual se divide 5cm de uma extremidade da fita adesiva no sentido do comprimento, colocando-se a extremidade intacta da fita sobre a ponta do nariz do paciente e enrolando cada uma das tiras de 5cm em torno da sonda ^(^ ^11^ ^)^ . 

 Já as opções de fixação comercial apresentam variação no *design* dos produtos em relação à forma, tipo de adesivo e associação com uma braçadeira ou trava, embora características semelhantes sejam perceptíveis. 

 Além desses, outros métodos de fixação são adotados de acordo com cada instituição. Apesar da variedade existente entre os métodos e da frequente utilização pelos enfermeiros, a segurança de tais tipos de fixação não possuem avaliação científica ^(^ ^1^ ^)^ e várias orientações de enfermagem relacionadas à fixação de SNG não são baseadas em evidências, mas em rituais e opiniões ^(^ ^12^ ^)^ . 

Ao considerar a escassez da literatura quanto à fixação da sonda nasogástrica, este estudo objetivou avaliar os métodos de fixação quanto a adesão, deslocamento e integridade da pele.

## Método

### Delineamento do estudo

 Trata-se de um estudo observacional e comparativo ^(^ ^13^ ^)^ , do tipo *ex* *vivo* , experimento em ambiente controlado, fora de um organismo vivo, envolvendo tecidos isolados ^(^ ^14^ ^)^ . Este tipo de estudo, normalmente aplicado à química e farmacologia, encontrou indicação nesta pesquisa por assegurar maior controle sobre possíveis variáveis de confusão. 

 As vantagens do estudo *ex* *vivo* correspondem à regra dos 3Rs, que são a substituição ( *replacement* ), uma vez que substituiu o uso de animais ou voluntários humanos na experimentação; a redução ( *reduction* ), relacionada ao menor número da amostra; e o refinamento ( *refinement* ), devido aos processos utilizados para maior aproximação da realidade ^(^ ^15^ ^)^ . 

### Local do estudo

O estudo foi realizado no laboratório de Biofísica da Universidade Católica de Pernambuco, no período de março a julho de 2022.

### Material para o experimento

 O material para o experimento foi constituído por: 06 placas de compensado com as seguintes dimensões: 30x40 cm e 10 mm de espessura; seis cortes de pele de porco ( *Sus scrofa domesticus* ) medindo 150x130 mm; 1 kg de pregos com cabeça com bitola de 0,9x9 mm; um rolo de linha 100% algodão; 30 unidades de sonda nasogástrica (sonda de Levine) calibre 14; uma unidade de esparadrapo impermeável 10 cm x 4,5 m composto por: algodão com resina acrílica impermeabilizante e massa adesiva à base de borracha natural, óxido de zinco e resina para as fixações das duas técnicas (A e B); 10 unidades de 3M fixador de tubos e sondas nasais, composto por: poliuretano laminado sobre não tecido de poliéster, adesivo acrílico hipoalergênico com *liner* de papel siliconado (Fixador C); um rolo de monofilamento nylon 0,30 mm x 100 m; três pesos de calibração para balança digital de aço inoxidável Mettler-Toledo, respectivamente de 50, 100 e 500 g; paquímetro digital com faixa de medição: 150 mm /0-6” polegadas e resolução de: 0.01 mm /0.0005 polegada. 

 Em cada bancada de 170 cm x 80 cm x 50 cm, foram colocadas duas placas expositoras de compensado, alinhadas uma ao lado da outra. Cada grupo de experimento correspondeu a duas placas expositoras da seguinte forma: Grupo A: fixador A ^(^ ^10^ ^)^ , Grupo B: fixador B ^(^ ^9^ ^)^ e Grupo C: Fixador C (fixador de tubos e sondas nasais comercial). 

 Sobre cada placa, colocou-se um corte de pele de porco, com a parte posterior (gordura) voltada para baixo, construindo-se, então, cinco narizes por corte (n=30), obedecendo às medidas nasais médias para a idade de 31-40 anos: comprimento nasal (n-prn) de 4,82 cm e 4,58 cm, altura nasal (n-sn): 5,22 cm e 4,97 cm e largura nasal (al-al): 3,51 cm e 3,10 cm, respectivamente, para os sexos masculino e feminino ^(^ ^16^ ^)^ ( Figura 1 ). Assim, o nariz experimental foi modelado e fixado através do uso de pregos e da linha de costura nas respectivas placas expositoras. 

 Para cada nariz experimental foram realizados dois furos, observando-se o diâmetro da narina humana de 10 e 12 mm ^(^ ^17^ ^)^ , utilizando-se um molde metálico e bisturi com lâmina n° 11. Cada nariz experimental foi separado do outro por uma distância de 3,5 mm. 

 Uma sonda nasogástrica foi inserida em cada nariz experimental, observando o comprimento de 50,7 cm, medida média de inserção da SNG relatada na literatura para o método de inserção da ponta do nariz ao lóbulo da orelha e deste até o apêndice xifoide de um humano adulto ^(^ ^18^ ^)^ . Esse comprimento foi marcado com uma fita de 1x2 cm de esparadrapo impermeável ao redor da sonda, alinhada à saída da narina, para servir de referência (marca de referência) à verificação de deslocamento. 

 Os fixadores foram aplicados de acordo com os respectivos procedimentos recomendados pela literatura para os fixadores A ^(^ ^10^ ^)^ , B ^(^ ^9^ ^)^ e do fixador C (comercial). A Figura 1 apresenta as etapas para construção do nariz experimental e os métodos de fixação de SNG utilizados no experimento: 

**Figura 1:** - Técnica de modelagem do nariz experimental e fixadores de SNG utilizados no experimento. Recife, PE, Brasil, 2022

Cada placa expositora foi coberta por um tecido de polipropileno de mesma dimensão, criando uma barreira com o objetivo de evitar a exposição das peles de porco a insetos indesejáveis.

A extremidade exterior de cada sonda foi ligada por um fio de nylon de 5 cm a pesos em aço inoxidável de 50, 100 e 500 g (Mettler-Toledo), sequencialmente, a cada 24 horas, de modo a simular as diversas situações de tração da SNG.

### Variáveis do estudo

 As variáveis consideradas neste estudo foram: 1) Método de fixação da sonda, categorizado como: fixador A ^(^ ^10^ ^)^ , fixador B ^(^ ^9^ ^)^ e fixador C; 2) A adesão, definida como a capacidade de fixação da sonda no nariz, na medida em que foi introduzida, variável dicotômica (fixação aderida ou não aderida a pele); 3) O deslocamento, definido como a diferença, em milímetros, entre a medida original da sonda no ponto de referência e a medida aferida em 12 e 24 horas após a introdução da sonda. Trata-se de uma variável racional; 4) A integridade da pele, avaliada como a presença de lesão após 24 horas da fixação da sonda. Para isso, utilizou-se o Sistema Internacional de Classificação de Lesões por Pressão NPUAP/EPUAP, classificando-as de acordo com o nível de perda de tecido em: perda parcial de espessura da derme, apresentando-se como úlcera aberta superficial e perda total de espessura do tecido quando a gordura subcutânea era visível ^(^ ^19^ ^)^ . Esta variável também foi avaliada pela área da lesão, medida pelo comprimento multiplicado pela largura e por 3,14 (variável racional); e 5) Tempo, categorizado pelos intervalos de 12 e 24 horas entre a fixação da sonda e a ocorrência dos eventos de interesse. 

### Coleta de dados

O roteiro de coleta de dados foi elaborado pelos pesquisadores e constou das variáveis anteriormente descritas. A coleta de dados foi realizada por uma das pesquisadoras.

Após 12 e 24 horas de cada exposição ao respectivo peso, verificou-se o deslocamento da SNG em relação à narina, utilizando um paquímetro digital, calibrado em milímetros, medindo a distância entre a marca de referência e a narina.

Ao final de 24 horas de experimento, foi removida a fixação, por um único pesquisador, usando removedor de adesivo em formato de lenço, composto por: Hexametildissiloxano, Octametiltrissiloxano, Ciclopentasiloxano, para remoção não traumática, diminuindo a força necessária para retirada do adesivo.

 Todas as lesões foram fotografadas, por uma câmera DSC-HX300 com zoom ótico de 50x e 20,4 MP (Sony Brasil Ltda.), sem uso de *flash* , e a medição da área da ferida foi realizada de acordo com o protocolo sistematizado na literatura ^(^ ^20^ ^)^ , ou seja: as fotos foram tiradas colocando uma régua descartável próxima ao ferimento em paralelo com a pele sã. Para minimizar o erro, a lente da câmera foi orientada paralelamente ao plano da ferida. Em seguida, uma segunda fotografia de todas as feridas foi obtida para garantir pelo menos uma fotografia de boa qualidade. 

As fotografias originais foram, então, copiadas para uma nova pasta, e os nomes dos arquivos (formato JPG) codificados para “A1” até “A10”, e assim respectivamente, para cada grupo do experimento, de modo a garantir o mascaramento do avaliador, que foi um enfermeiro estomaterapeuta, com experiência de 40 anos com cuidados de pessoas com lesão de pele, que não teve informação sobre o tipo de fixador utilizado em cada grupo.

 As fotografias digitais foram visualizadas com o *software* ImageJ 1.45s ( *National Institutes of Health* , Rockville, MD). 

### Tratamento e análise de dados

Para o tratamento estatístico, foram utilizados a média e desvio padrão (DP) dos deslocamentos ocorridos e da área das lesões. Foi calculado o teste Qui-quadrado de independência para verificar a existência de associação entre os tipos de fixadores e a capacidade de adesão, através da razão de verossimilhança. Para verificar a normalidade da distribuição dos dados, aplicou-se o teste de Shapiro-Wilk (normalidade: p> 0,05). Para comparação de médias entre dois grupos de fixadores quanto ao deslocamento da SNG foi utilizado o teste t-Student e, a análise de variância (ANOVA) foi calculada para os três grupos de fixadores quanto à área da lesão. Para fins de análise, observou-se o nível de significância de 5%.

### Aspectos éticos

 Ao considerar o tipo de estudo realizado [estudo *ex vivo* no qual foram utilizados cortes de pele de porco ( *Sus scrofa domesticus* ) fora de um organismo vivo (tecidos isolados) para simulação do nariz humano], dispensa-se a avaliação por Comitê de Ética. 

## Resultados

 Nas primeiras 12 horas de uso da SNG, com uma tração de 50 e 100 g, todos os fixadores permaneceram aderidos, mas quando submetidos a uma tração de 500g, houve descolamento em 10% dos fixadores A, e 50% dos fixadores B. Da mesma forma, após 24 horas de exposição, os fixadores mantiveram-se aderidos com as trações menores e, quando a SNG foi submetida a uma tração de 500 g, observou-se descolamento em 10% dos fixadores A e 70% dos fixadores B. Não se observou o descolamento associado ao fixador C quando exposto aos três pesos nem aos tempos de 12 e 24 horas ( Tabela 1 ). 

 Nota-se a maior frequência de descolamento durante o uso do fixador B quando comparado aos outros, aumentando a diferença, inclusive quanto ao maior tempo de exposição. O teste Qui-quadrado de independência mostrou a existência de associação entre o descolamento dos fixadores quanto a tração aplicada por 12 [χ ^2^ (2)= 9,660; p=0,008] e 24 horas [χ ^2^ (2)= 16,076; p<0,001]. 

**Tabela 1:** - Associação da adesão do fixador quanto à submissão de 500 g de tração por 12 e 24 horas. Recife, PE, Brasil, 2022

**Variável**	**Adesão 12 horas**		**p-valor**	**Adesão 24 horas**		**p-valor** ^*^
	**Sim** ^(%)^	**Não** ^(%)^		**Sim** ^(%)^	**Não** ^(%)^	
Fixador A	9 (90,0)	1(10,0)		9 (90,0)	1 (10,0)	
Fixador B	5 (50,0)	5 (50,0)	0,008	3 (30,0)	7 (70,0)	<0,001
Fixador C	10 (100,0)	0 (0,0)		10 (100,0)	0 (0,0)	

^*^ p-valor = Nível de significância

No que diz respeito ao deslocamento da SNG quando submetida à tração, verificou-se evento similar ao ocorrido quanto à adesão, ou seja, as trações de 50 e de 100 g não causaram o deslocamento da SNG nas 12 e 24 horas de observação, somente a tração de 500 g determinou um deslocamento nas 12 horas em 40% dos fixadores A e B (X: 34,04 mm; DP: 4,8) e um deslocamento em 56,7% dos mesmos dispositivos nas 24 horas de observação (X: 34,78 mm; DP: 4,9).

 Dentre as três fixações analisadas, a fixação C não apresentou deslocamento mesmo quando submetida à tração de 500 g. O teste t-Student realizado para comparação das médias de deslocamento das fixações A e B mostrou a igualdade de variâncias, conforme valores de p >0,05 nos dois períodos de exposição ( Tabela 2 ). 

 Quanto à integridade da pele ( Figura 2 ), ao final do experimento observou-se a ocorrência de lesões em todos os narizes expostos aos fixadores, dos quais 90% caracterizaram-se por perda total de espessura do tecido e 10% por perda parcial. As maiores frequências de lesão ocorreram para os Grupos A e C com diferença estatística significante (p= 0,036) 

**Tabela 2:** - Comparação entre as médias de deslocamento entre os fixadores A e B. Recife, PE, Brasil, 2022

**Deslocamento**	**Fixador**	**n** ^*^	**X mm** ^†^	**DP** ^‡^	**t** ^§^	**p-valor** ^||^
Deslocamento nas 12 horas	Fixador A	10	72,003	56,3959	1,957	0,066
	Fixador B	10	30,120	37,4081		
Deslocamento nas 24 horas	Fixador A	10	64,056	46,0694	1,356	0,192
	Fixador B	10	40,295	30,7552		

^*^ n = Amostra;

^†^ X mm = Média do deslocamento em milímetros;

^‡^ DP = Desvio-padrão;

^§^ t = t-Student;

^||^ p-valor = Nível de significância

**Figura 2:** - Lesões segundo o grupo de fixação da sonda nasogástrica. Recife, PE, Brasil, 2022

 A ANOVA de 1 via mostrou que existe efeito do tipo de fixador sobre a área da lesão [F (2,27)= 8,88; p: 0,001]. O *post-hoc* de Bonferroni mostrou que em média a área da lesão das sondas fixadas com o Grupo A é diferente daquelas fixadas pelo Grupo B, assim como a média da área da lesão das sondas fixadas com o Grupo C é diferente das que foram fixadas pelo Grupo B ( Tabela 3 ). 

**Tabela 3:** - Comparação das médias das áreas das lesões segundo o grupo de fixação da sonda nasogástrica. Recife, PE, Brasil, 2022

**Grupos**	**X** ^*^	**DP** ^†^	**F** ^‡^	**p-valor** ^§^
Fixador A	60,43	5,8		
Fixador B	22,90	19,3	8,88	0,001
Fixador C	68,84	40,1		

^*^ X = Média das áreas das lesões;

^†^ DP = Desvio-padrão;

^‡^ F = Teste de Levene;

^§^ p-valor = Nível de significância

## Discussão

A sondagem nasogástrica é um procedimento antigo e comumente realizado em pessoas de todas as idades, seja na atenção domiciliar, seja nos cuidados hospitalares de média ou de alta complexidade e embora pareça uma técnica simples, pode estar relacionada a complicações graves que refletem na segurança do paciente.

 Os eventos adversos relacionados à etapa da inserção e de manutenção da SNG correspondem principalmente à colocação ou migração intrapulmonar com administração de alimento, medicamento ou fluido; pneumotórax e colocação ou migração intra-esofágica, que predispõe à pneumonia por aspiração ^(^ ^20^ ^-^ ^21^ ^)^ . 

 Embora seja possível estimar a incidência de eventos adversos em termos de número de SNGs, os dados sobre número de sondas utilizadas para alimentação, hidratação, administração de medicamentos, lavagem gástrica e drenagem ainda são desconhecidos em muitos países. Nos EUA, aproximadamente um milhão de SNG/Sondas nasoentéricas (SNE) são utilizadas no tratamento de adultos e crianças a cada ano, e estima-se a ocorrência de eventos adversos em 1 a 3% desses procedimentos ^(^ ^21^ ^)^ . A Inglaterra apresenta uma incidência estimada de 1 evento adverso em 10.000 inserções ou 0,01% ^(^ ^20^ ^)^ . 

Além desses, as técnicas e/ou os dispositivos usados para fixação da sonda nasogástrica, de acordo com o tempo de permanência, a ação da gravidade e a tração causada tanto pelas conexões para procedimentos de lavagem ou drenagem, como por ação mecânica, realizada pelo próprio paciente e ou cuidador, no sentido de evitar o desconforto causado e/ou por questões culturais, podem, em tese, causar o seu deslocamento, aumentando o risco para os eventos anteriormente citados.

 Quanto ao deslocamento da SNG, este estudo controlado buscou verificar, através de comparação, a resistência de três tipos de fixadores, tomando por premissa que, apesar da existência de fixadores industrializados, não existem evidências científicas que aprovem o seu uso e, o custo de aquisição muitas vezes está distante dos recursos dos sistemas públicos de saúde. Além disso, ao longo do tempo, enfermeiros e outros profissionais de saúde tem desenvolvido várias técnicas de fixação, que também carecem de evidências para fundamentar seu uso ^(^ ^22^ ^)^ . 

 De forma geral, os três tipos de fixadores apresentaram uma boa capacidade de adesão. Apenas o fixador B ^(^ ^9^ ^)^ apresentou desempenho inferior quando exposto à tração de 500g, comparado aos outros dois fixadores. Nesta técnica, uma fita adesiva, medindo 13 centímetros de comprimento por um centímetro de largura, envolve completamente a SNG e, em seguida, é fixada sobre o lábio superior. A menor área do lábio e sua maior mobilidade quando comparada ao nariz, a princípio, parecem justificativas plausíveis para esse resultado. 

O fixador C (comercial) apresentou maior capacidade de adesão, evitando deslocamento da SNG quando comparado aos fixadores manufaturados (fixador A e B), para os quais não se encontrou diferença quanto às médias de deslocamento das SNGs.

 Em média, observou-se um deslocamento de 52,17 mm nas fixações A ^(^ ^10^ ^)^ e B ^(^ ^9^ ^)^ . O comprimento seguro de inserção da SNG depende da técnica de mensuração utilizada e da altura corporal, estimando-se, em média, para adulto 55-65 cm, quando utilizada a técnica convencional, do nariz ao lóbulo da orelha e desta até o apêndice xifoide ^(^ ^23^ ^)^ . Logo, o deslocamento encontrado em nosso experimento sugere um risco para efeitos adversos, com possível migração da sonda para o esôfago o que pode ocasionar refluxo e broncoaspiração. 

 O risco de aspiração do conteúdo gástrico do esôfago para os pulmões, assim como a associação entre pneumonia por aspiração e deslocamento esofágico não são conhecidos, no entanto, essa complicação é potencialmente fatal e também deve ser o assunto de preocupação com a segurança do procedimento de sondagem nasogástrica ^(^ ^20^ ^)^ . 

 Quanto à integridade da pele, nosso estudo mostrou que todos os fixadores testados causaram lesão com perda total da espessura da pele, com maiores frequências para o fixador A ^(^ ^10^ ^)^ e para o fixador C. Na verdade, ambos são aderidos ao septo nasal, numa área consideravelmente maior do que aquela utilizada na técnica pelo fixador B. 

 Apesar da limitada informação a respeito de lesões relacionadas a dispositivos médicos, estudos têm mostrado prevalência de 8 a 9,4% das lesões por pressão associadas a sonda nasogástrica ^(^ ^20^ ^,^ ^24^ ^-^ ^25^ ^)^ . A composição dos adesivos dos fixadores quando expostos ao tempo, as condições da pele e a tração podem, em tese, sugerir uma explicação para essas lesões. 

O presente estudo contribui para o avanço do conhecimento científico na área de enfermagem tanto pelo método utilizado, que se mostrou útil para testar variáveis como adesão, deslocamento e ocorrência de lesão de pele associadas à fixação de sonda nasogástrica, assim como também pelos resultados inéditos que permitiram maior reflexão para escolha da fixação, elaboração de protocolos e de estratégias de prevenção de efeitos adversos como a lesão de pele. Entretanto, assume-se como limitação o fato de que outras variáveis podem influenciar na interface entre o tipo de fixação e a adesão, deslocamento e lesão, recomendando-se que novos estudos in vivo sejam realizados para melhor elucidar os fenômenos aqui descritos.

## Conclusão

Os resultados aqui encontrados são inéditos e acrescentam ao conhecimento sobre questões relacionadas aos fixadores de sonda nasogástrica.

O fixador B, desenvolvido para maior conforto do paciente, tendo em vista a pequena área de fixação, esteve associado a falhas de adesão, enquanto o fixador comercial (C) apresentou maior capacidade para isto.

 Os dois fixadores manufaturados estiveram associados a deslocamentos consideráveis da SNG, o que pode expor o paciente a maior risco de eventos adversos como refluxo gastresofágico e broncoaspiração. Entretanto, no que diz respeito à integridade da pele, os resultados mostraram que tanto o fixador C como o fixador A ^(^ ^10^ ^)^ estiveram associados a lesões de perda total da espessura da pele. 

Os resultados apresentados atestam complicações relacionadas aos fixadores da sonda nasogástrica e chamam atenção para complexidade do procedimento. Diante disso, o conhecimento e habilidade do enfermeiro na inserção e na realização dos cuidados subsequentes são importantes para garantir a segurança do paciente.
